# Supplementary figures and images for: Engineered ketocarotenoid biosynthesis in the polyextremophilic red microalga Cyanidioschyzon merolae 10D
Source: Metab Eng Commun. 2023 Jun 26;17:e00226. doi: 10.1016/j.mec.2023.e00226 (PMC10336515; doi:10.1016/j.mec.2023.e00226)

Genome – Chromosome 4 (NC\_010130.1):

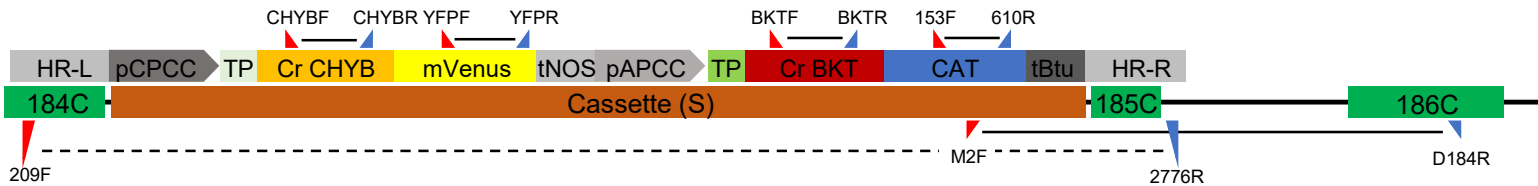

Plasmid

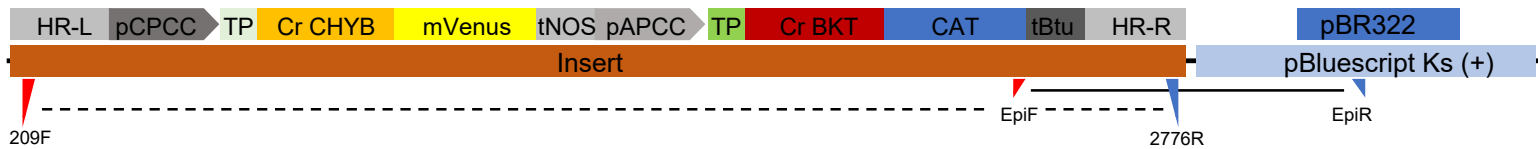

Genome – Chromosome NC\_004799:

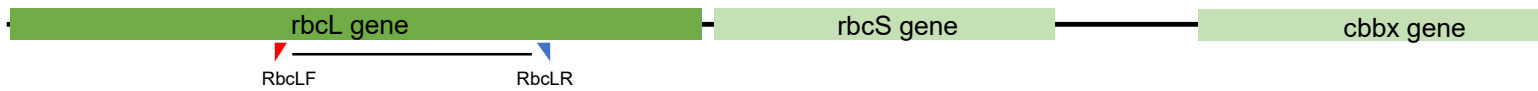

Supplement: Multimedia component 1 [file mmc1.pdf]
